# A Single-Amino-Acid Substitution at Position 225 in Hemagglutinin Alters the Transmissibility of Eurasian Avian-Like H1N1 Swine Influenza Virus in Guinea Pigs

**DOI:** 10.1128/JVI.00800-17

**Published:** 2017-10-13

**Authors:** Zeng Wang, Huanliang Yang, Yan Chen, Shiyu Tao, Liling Liu, Huihui Kong, Shujie Ma, Fei Meng, Yasuo Suzuki, Chuanling Qiao, Hualan Chen

**Affiliations:** a State Key Laboratory of Veterinary Biotechnology, Harbin Veterinary Research Institute, Chinese Academy of Agricultural Sciences (CAAS), Harbin, People's Republic of China; b College of Life and Health Sciences, Chubu University, Aichi, Japan; University of Southern California

**Keywords:** EAH1N1, transmissibility, genetic basis

## Abstract

Efficient transmission from human to human is the prerequisite for an influenza virus to cause a pandemic; however, the molecular determinants of influenza virus transmission are still largely unknown. In this study, we explored the molecular basis for transmission of Eurasian avian-like H1N1 (EAH1N1) swine influenza viruses by comparing two viruses that are genetically similar but differ in their transmissibility in guinea pigs: the A/swine/Guangxi/18/2011 virus (GX/18) is highly transmissible by respiratory droplet in guinea pigs, whereas the A/swine/Heilongjiang/27/2012 virus (HLJ/27) does not transmit in this animal model. We used reverse genetics to generate a series of reassortants and mutants in the GX/18 background and tested their transmissibility in guinea pigs. We found that a single-amino-acid substitution of glycine (G) for glutamic acid (E) at position 225 (E225G) in the HA1 protein completely abolished the respiratory droplet transmission of GX/18, whereas the substitution of E for G at the same position (G225E) in HA1 enabled HLJ/27 to transmit in guinea pigs. We investigated the underlying mechanism and found that viruses bearing 225E in HA1 replicated more rapidly than viruses bearing 225G due to differences in assembly and budding efficiencies. Our study indicates that the amino acid 225E in HA1 plays a key role in EAH1N1 swine influenza virus transmission and provides important information for evaluating the pandemic potential of field influenza virus strains.

**IMPORTANCE** Efficient transmission among humans is a prerequisite for a novel influenza virus to cause a human pandemic. Transmissibility of influenza viruses is a polygenic trait, and understanding the genetic determinants for transmissibility will provide useful insights for evaluating the pandemic potential of influenza viruses in the field. Several amino acids in the hemagglutinin (HA) protein of influenza viruses have been shown to be important for transmissibility, usually by increasing virus affinity for human-type receptors. In this study, we explored the genetic basis of the transmissibility difference between two Eurasian avian-like H1N1 (EAH1N1) swine influenza viruses in guinea pigs and found that the amino acid glutamic acid at position 225 in the HA1 protein plays a critical role in the transmission of EAH1N1 virus by increasing the efficiency of viral assembly and budding.

## INTRODUCTION

Human and animal influenza viruses are very closely related. The H2N2 virus and H3N2 virus that caused the 1957 and 1968 influenza pandemics, respectively, are reassortants of human influenza viruses and avian influenza viruses (1), and the H1N1 virus that caused the human influenza pandemic in 2009 is a swine influenza reassortant, with one parental strain containing the PB1 gene from a human influenza virus (2). As of the end of March 2017, the H5N1 and H7N9 avian influenza viruses had caused 858 and 1,307 human infections, with 453 and 489 deaths, respectively (http://www.who.int/influenza), representing clear pandemic threats. Recent studies indicate that the naturally occurring Eurasian avian-like H1N1 (EAH1N1) swine influenza viruses (SIVs) and H3N8 and H9N2 avian influenza viruses can transmit in ferrets via respiratory droplet (3–5), revealing the pandemic potential of these overlooked animal influenza viruses.

Efficient transmission from human to human is the prerequisite for influenza virus to cause a pandemic. Ferret and guinea pig are two commonly used animal models to evaluate the transmissibility of animal influenza viruses in humans (6–9). By using these two models, researchers have identified a series of important genetic markers of influenza virus transmissibility. Tumpey et al. reported that a 2-amino-acid change, D190E and D225G, in the viral hemagglutinin (HA) protein abolishes the transmission of 1918/H1N1 influenza virus in ferrets (10). The amino acids at positions 222 and 226 of HA are critical for the 2009/H1N1 virus to bind to human-type receptors and transmit among mammals (11, 12), and PB2 271A facilitates the mutation of R to Q at position 226 of the HA of 2009/H1N1 viruses (11). Amino acids at other positions of HA and PB2 have also been shown to be critical for transmission of H5N1 influenza viruses in mammals (13–15). Zhang et al. reported that an H5N1 virus that derived its PA or NS gene from the 2009/H1N1 virus becomes transmissible in guinea pigs, indicating that gene constellations also play an important role in influenza virus transmission (16).

We previously characterized EAH1N1 swine influenza viruses isolated from pigs in China and found that these viruses have different transmissibility in ferrets (3). When we evaluated the transmissibility of these viruses in guinea pigs, we found that two viruses, A/swine/Guangxi/18/2011 (GX/18) and A/swine/Heilongjiang/27/2012 (HLJ/27), were genetically similar but differed in their transmissibility: GX/18 was highly transmissible via respiratory droplet, whereas HLJ/27 could not transmit in this animal model. In this study, we explored the underlying mechanism of this difference in transmissibility.

## RESULTS

### Virus rescue and transmissibility testing.

We cloned the cDNAs of each full-length RNA segment of the GX/18 and HLJ/27 viruses into a viral RNA (vRNA)-mRNA bidirectional expression plasmid (pBD) as described previously (17) and by using the gene segment-specific primers shown in Table 1. All of the constructs were completely sequenced to ensure the absence of unwanted mutations. By using these plasmids, we rescued the GX/18 and HLJ/27 viruses, designated rGX/18 and rHLJ/27, respectively; grew them in 10-day-old specific-pathogen-free (SPF) embryonated chicken eggs; and compared their respiratory droplet transmission with that of the wild-type GX/18 and HLJ/27 viruses in guinea pigs.

**TABLE 1:** Primers used for pBD cDNA construction and for introducing mutations into the HA genes of the mutant viruses

Purpose	Primers (5′–3′)^*a*^	
	Forward	Reverse
PB2 amplification	CCAGCAAAAGCAGGTCAAATATATTCAA	TTAGTAGAAACAAGGTCGTTTTTAA
PB1 amplification	CCAGCAAAAGCAGGCAAACCATTTGA	TTAGTAGAAACAAAGGCATTTTTTCATGA
PA amplification	CCAGCAAAAGCAGGTACTGAT	TTAGTAGAAACAAGGTACTTTTTTGGACAG
HA amplification	CCAGCAAAAGCAGGGGAAAATT	TTAGTAGAAACAAGGGTGTTTTT
NP amplification	CCAGCAAAAGCAGGGTAGATAATCACTCA	TTAGTAGAAACAAGGGTATTTTTCTT
NA amplification	CCAGCAAAAGCAGGAGTTTAAAATG	TTAGTAGAAACAAGGAGTTTTTTG
M amplification	CCAGCAAAAGCAGGTAGATAT	TTAGTAGAAACAAGGTAGTTTTTTACTC
NS amplification	CCAGCAAAAGCAGGGTGACAAA	TTAGTAGAAACAAGGGTGTTTTTTAT
GX/18-HA1-V15I mutation	AGCTGACACCATTTGT**A**TAGGCTAC	**T**ACAAATGGTGTCAGCTTTTAGTGC
GX/18-HA1-A103T mutation	ACCCCGGAGAATTC**A**CTGATTATGA	**T**GAATTCTCCGGGGTAGCATGCTCC
GX/18-HA1-S138A mutation	ACCAGAGGTACCACAGTT**G**CATGCT	**C**AACTGTGGTACCTCTGGTGGTATC
GX/18-HA1-E225G mutation	AGACCTAAAGTCAGAG**G**ACAAGCAG	**C**CTCTGACTTTAGGTCTTGCTACTA
GX/18-HA1-T235I mutation	GAATGAATTATTACTGGA**T**ACTGTTAG	**A**TCCAGTAATAATTCATTCTGCCTG
GX/18-HA1-H256Y mutation	TAATAGCACCATGG**T**ACGCATTTGC	**A**CCATGGTGCTATTAAATTCCCAGT
GX/18-HA1-R269 M mutation	AGTTCTGGAATTATGA**T**GTCGGATG	**A**TCATAATTCCAGAACTAGAGCCTT
GX/18-HA2-R27Q mutation	GGTATGGATATCACCATC**A**AAATGA	**T**GATGGTGATATCCATACCATCCAT
GX/18-HA2-L160P mutation	TGGCACATACAATTATC**C**CAAATAT	**G**GATAATTGTATGTGCCATTCTTTA
GX/18-HA2-I183V mutation	ACTAGAATCAATGGGA**G**TTCACCAG	**C**TCCCATTGATTCTAGTTTCACACC
HLJ/27-HA1-G225E mutation	CAAGACCTAAAGTCAGAG**A**ACAAGC	**T**CTCTGACTTTAGGTCTTGCTACTA

a Nucleotides that were changed are underlined and in boldface.

Three guinea pigs were inoculated intranasally (i.n.) with 10^6^ 50% egg infective doses (EID_50_) of each virus and then housed separately in solid stainless-steel cages within an isolator. Twenty-four hours later, three naive animals were placed in adjacent cages. Nasal washes were collected from all of the animals at 2-day intervals beginning 2 days postinoculation (p.i.) (1 day postexposure) for the detection of virus shedding. Sera were collected from all the animals on day 21 p.i. for hemagglutinin inhibition (HI) antibody detection. Respiratory droplet transmission was confirmed by detecting virus in the nasal washes or seroconversion at the end of the 3-week observation period.

**FIG 1:**
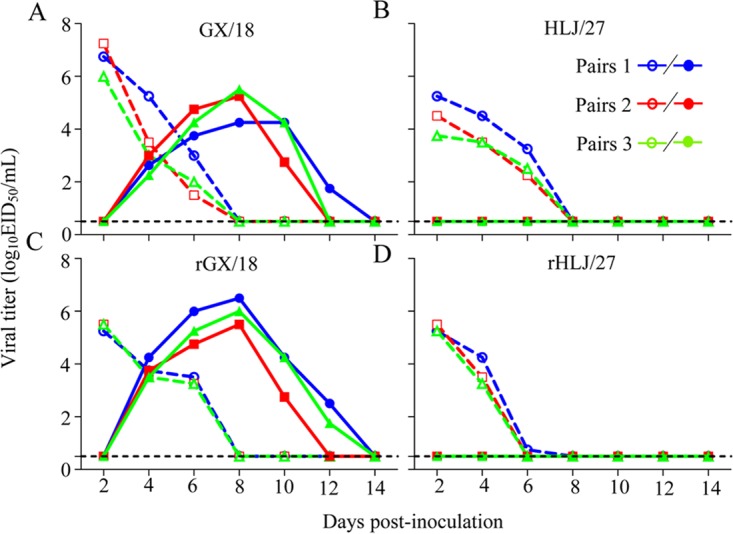
Respiratory droplet transmission of EAH1N1 SIVs in guinea pigs. Groups of three guinea pigs were inoculated intranasally with 10^6^ EID_50_ of test viruses. Twenty-four hours later, three naive guinea pigs were placed in adjacent cages. Nasal washes were collected at 2-day intervals, beginning 2 days p.i. (1 day postexposure), and assessed for virus shedding. Each line represents the virus titer from an individual animal. The dashed lines indicate the values from the inoculated animals; the solid lines indicate the values from the exposed animals. The dashed black lines indicate the lower limit of detection.

Virus was detected in all of the virus-inoculated animals between days 2 and 6 p.i. (Fig. 1). However, in the exposed groups, virus was detected only in the three animals exposed to guinea pigs infected with GX/18 or rGX/18 (Fig. 1A and C and Table 2); the animals exposed to the guinea pigs infected with HLJ/27 or rHLJ/27 showed no signs of infection (Fig. 1B and D and Table 2). Seroconversion occurred in all the inoculated animals and in the GX18- and rGX/18-exposed animals (Table 2). These results indicate that the rescued viruses maintained the same transmissibility phenotype as the wild-type viruses.

**TABLE 2:** Seroconversion of guinea pigs inoculated with or exposed to EAH1N1 reassortants and mutants

Virus	Passage history^*a*^	No. of animals with virus shedding/total		No. of animals with seroconversion/total (HI titer range)	
		Inoculated	Exposed	Inoculated	Exposed
GX/18	E3	3/3	3/3	3/3 (320–640)	3/3 (320–640)
HLJ/27	E2	3/3	0/3	3/3 (320–640)	0/3
rGX/18	E1	3/3	3/3	3/3 (640–1,280)	3/3 (320–640)
rHLJ/27	E1	3/3	0/3	3/3 (1,280–2,560)	0/3
rGX/18-HLJPB2	E1	3/3	2/3	3/3 (320–640)	3/3 (80–320)
rGX/18-HLJPB1	E1	3/3	3/3	3/3 (1,280–2,560)	3/3 (640–1,280)
rGX/18-HLJPA	E1	3/3	3/3	3/3 (2,560–5,120)	3/3 (640–1,280)
rGX/18-HLJHA	E1	3/3	1/3	3/3 (640–1,280)	1/3 (640)
rGX/18-HLJNP	E1	3/3	3/3	3/3 (320–640)	3/3 (80–320)
rGX/18-HLJNA	E1	3/3	3/3	3/3 (640–1,280)	3/3 (320–640)
rGX/18-HLJM	E1	3/3	2/3	3/3 (1,280)	3/3 (320–640)
rGX/18-HLJNS	E1	3/3	3/3	3/3 (640–1,280)	3/3 (320–640)
rGX/18-HLJHA-repeat	E1	3/3	1/3	3/3 (640–5,120)	1/3 (2560)
rHLJ/27-GXHA	E1	3/3	2/3	3/3 (640–1,280)	3/3 (1,280–2,560)
rGX18-HA1-V15I	E1	3/3	3/3	3/3 (640–1280)	3/3 (160–640)
rGX18-HA1-A103T	E1	3/3	2/3	3/3 (640)	3/3 (160)
rGX18-HA1-S138A	E1	3/3	3/3	3/3 (640–2,560)	3/3 (640)
rGX/18-HA1-E225G	E1	3/3	0/3	3/3 (320–640)	0/3
rGX18-HA1-T235I	E1	3/3	2/3	3/3 (2,560–5,120)	3/3 (640–1,280)
rGX18-HA1-H256Y	E1	3/3	2/3	3/3 (320–640)	3/3 (40–320)
rGX18-HA1-R269 M	E1	3/3	2/3	3/3 (320–640)	3/3 (320–640)
rGX18-HA2-R27Q	E1	3/3	3/3	3/3 (320–640)	3/3 (160–320)
rGX18-HA2-L160P	E1	3/3	3/3	3/3 (1,280)	3/3 (160)
rGX18-HA2-I183V	E1	3/3	3/3	3/3 (640–1,280)	3/3 (160–320)
rHLJ27-HA1-G225E	E1	3/3	2/3	3/3 (1,280)	3/3 (80–320)

a E, chicken embryo.

### The HA gene plays a major role in the difference in transmissibility in guinea pigs between the GX/18 and HLJ/27 viruses.

**FIG 2:**
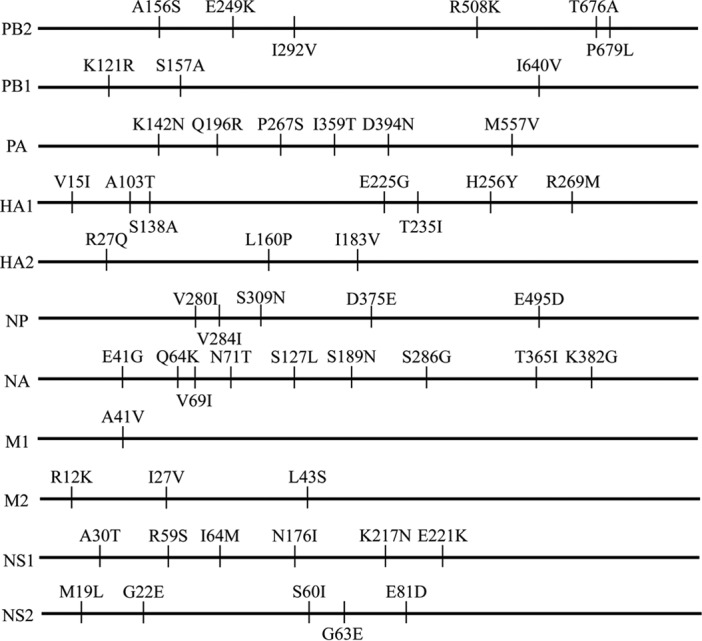
Amino acid differences between the GX/18 and HLJ/27 viruses. The amino acid differences between the two viruses are shown as single letters at the indicated positions. Each amino acid of GX/18 is shown before the number of the position, and each amino acid of HLJ/27 is shown after the number of the position.

**FIG 3:**
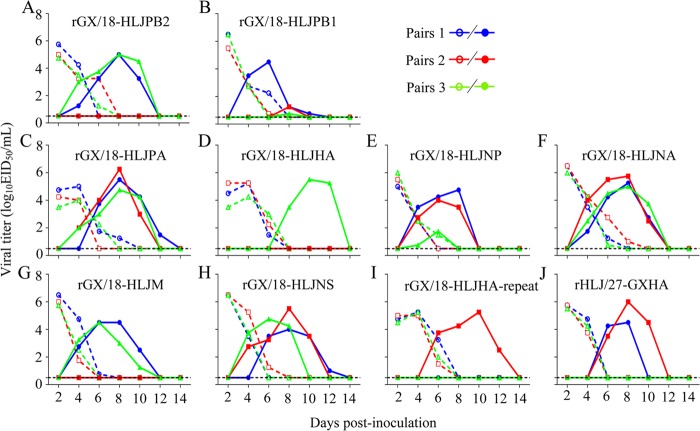
Respiratory droplet transmission of the reassortant viruses in guinea pigs.

The GX/18 and HLJ/27 viruses differ by 54 amino acids in their 11 proteins (Fig. 2). To identify the genes that contribute to the transmission of these viruses in guinea pigs, we generated a series of reassortant viruses by using reverse genetics as described previously (17). We used GX/18 as the backbone to generate eight reassortant viruses, designated rGX/18-HLJPB2, rGX/18-HLJPB1, rGX/18-HLJPA, rGX/18-HLJHA, rGX/18-HLJNP, rGX/18-HLJNA, rGX/18-HLJM, and rGX/18-HLJNS, and tested their transmissibility in guinea pigs. Five viruses, rGX/18-HLJPB1, rGX/18-HLJPA, rGX/18-HLJNP, rGX/18-HLJNA, and rGX/18-HLJNS, transmitted from the infected animals to all three exposed guinea pigs (Fig. 3B, C, E, F, and H and Table 2); two viruses, rGX/18-HLJPB2 and rGX/18-HLJM, transmitted from the infected animals to two of three exposed guinea pigs (Fig. 3A and G and Table 2). Seroconversion was detected in all of the guinea pigs inoculated with or exposed to these seven reassortant viruses (Table 2). However, the virus rGX/18-HLJHA, which carries the HA gene of HLJ/27, transmitted to only one of the three exposed guinea pigs, as evidenced by both virus isolation and serological testing (Fig. 3D and Table 2).

To investigate how the HA gene of the GX/18 virus affects the transmissibility of the HLJ/27 virus, we generated a reassortant virus, rHLJ/27-GXHA, by introducing the HA gene of GX/18 into the background of the HLJ/27 virus and tested its transmissibility in guinea pigs. The rHLJ/27-GXHA virus was detected in all three directly infected animals and in two of the three exposed animals (Fig. 3J and Table 2); seroconversion was detected in all of the inoculated and exposed animals (Table 2). These results indicate that HA plays an important role in the difference in transmissibility between the two viruses.

### The amino acid glutamic acid (E) at position 225 of HA is critical for the transmissibility of the GX/18 virus in guinea pigs.

**FIG 4:**
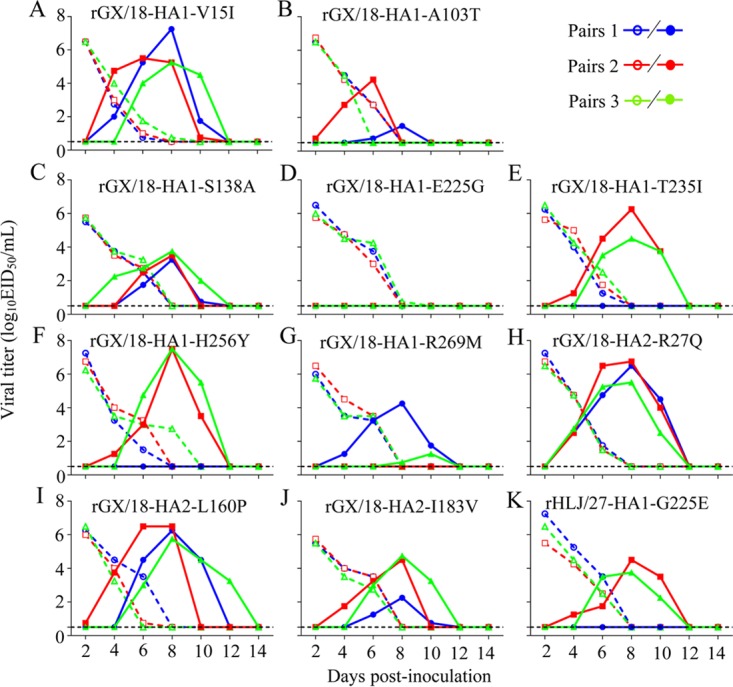
Respiratory droplet transmission of the mutant viruses in guinea pigs.

The HA proteins of the GX/18 and HLJ/27 viruses differ by 10 amino acids, 7 of which are at positions 15, 103, 138, 225, 235, 256, and 269 (H3 numbering) of the HA1 protein, whereas the other three are at positions 27, 160, and 183 of the HA2 protein (Fig. 2). To determine which amino acid(s) contributes to the transmission of the GX/18 virus, we generated 10 mutants in the rGX/18 background, each of which carried a mutation in HA that was present in the HLJ/27 virus at that position, and tested their transmissibility in guinea pigs. Five viruses bearing mutations at positions 15 and 138 of HA1 and at 27, 160, and 183 of HA2 transmitted to all three exposed guinea pigs (Fig. 4A, C, H, I, and J and Table 2); four viruses bearing mutations at positions 103, 235, 256, and 269 of HA1 transmitted to two of three exposed guinea pigs (Fig. 4B, E, F, and G and Table 2). Seroconversion was detected in all three animals exposed to the animals infected with each of these nine viruses (Table 2). One virus, rGX/18-HA1-E225G, did not transmit to any of the exposed animals (Fig. 4D and Table 2), indicating that the E225G mutation of HA1 eliminated the transmission of the GX/18 virus in guinea pigs.

We then introduced the G225E mutation of HA1 into the HLJ/27 virus and tested the transmissibility of the mutant in guinea pigs. We detected the rHLJ/27-HA1-G225E virus in two of the three exposed animals (Fig. 4K and Table 2), and seroconversion was detected in all three exposed animals (Table 2). These results indicate that the amino acid at position 225 of HA1 contributes to the difference in transmissibility between the GX/18 and HLJ/27 viruses in guinea pigs.

**FIG 5:**
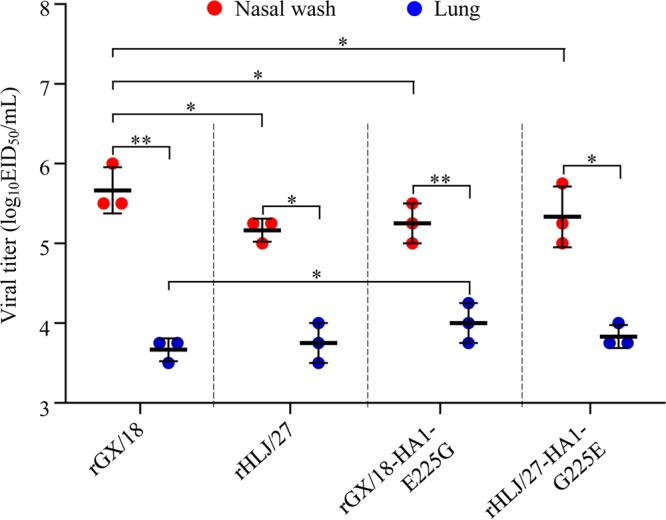
Replication of EAH1N1 SIVs in guinea pigs. Groups of three guinea pigs were i.n. inoculated with 10^6^ EID_50_ of the indicated virus and euthanized on day 3 p.i.; nasal washes and lungs were collected for virus titration in eggs. The results are presented as means ± standard deviations for three guinea pigs in each group. The values were statistically analyzed by using a one-tailed paired *t* test. *, *P* < 0.05; **, *P* < 0.01.

We also tested the replication of the rGX/18, rHLJ/27, rGX/18-HA1-E225G, and rHLJ/27-HA1-G225E viruses in guinea pigs. As shown in Fig. 5, all of the viruses replicated in the nasal tracts with titers significantly higher than those in the lungs of guinea pigs. Of note, the viral titer in the nasal tracts of the rGX/18-inoculated animals was significantly higher than that in the nasal tracts of the animals inoculated with the other three viruses.

### The viruses recovered from the guinea pigs that were exposed to the rGX/18-HLJHA virus contained a D24N mutation in the HA1 gene.

Although the rGX/18-HA1-E225G variant did not transmit in guinea pigs, the rGX/18-HLJHA virus transmitted to one of the three exposed guinea pigs, and this finding was reproducible (Fig. 3D and I and Table 2). When we sequenced the whole genome of the rGX/18-HLJHA virus that was recovered from the animals in the transmission studies, we found that a D24N mutation consistently occurred in the HA1 gene of the rGX/18-HLJHA virus and that this mutation was not detected in any samples that we collected from rGX/18-HLJHA-infected animals. These results suggest that the D24N mutation in HA may play a role in the transmission of the rGX/18-HLJHA virus.

### The amino acid at position 225 of HA affects viral replication in MDCK cells.

**FIG 6:**
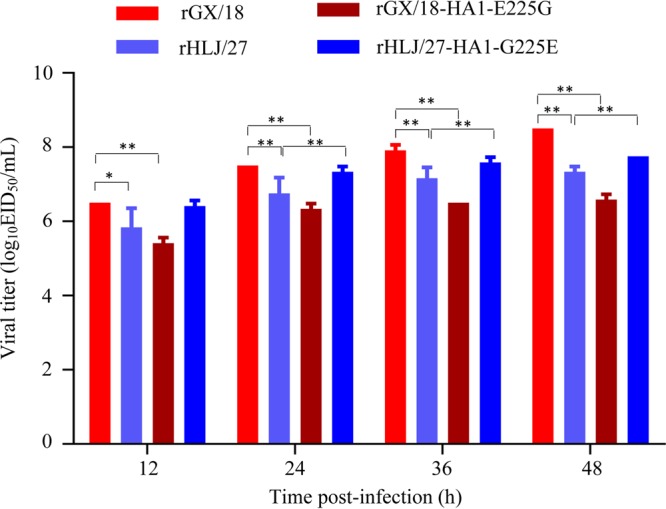
Multicycle replication of EAH1N1 SIVs in MDCK cells. MDCK cell monolayers were inoculated at an MOI of 0.01 with test viruses; cell supernatants were collected at the indicated time points and titrated in eggs. *, *P* < 0.05; **, *P* < 0.01. The error bars indicate standard deviations.

We compared the multicycle growth of rGX/18 and rHLJ/27 in Madin-Darby canine kidney (MDCK) cells and found that the rGX/18 virus grew more rapidly than did the rHLJ/27 virus and that the titers of rGX/18 were significantly higher than those of rHLJ/27 at 24, 36, and 48 h postinfection (Fig. 6). We then investigated the contribution of the amino acid change at position 225 in HA1 to the replication of these two viruses. Titers of rGX/18-HA1-E225G were significantly lower than those of rGX/18 at all four time points postinfection, whereas the replication level of rHLJ/27-HA1-G225E was significantly increased at 24, 36, and 48 h postinfection (Fig. 6). These results indicate that the amino acid at position 225 of HA1 affects the replication of these two EAH1N1 viruses.

### The mutation at position 225 of HA alters the receptor-binding affinity of the GX/18 and HLJ/27 viruses but does not switch the receptor-binding preference.

**FIG 7:**
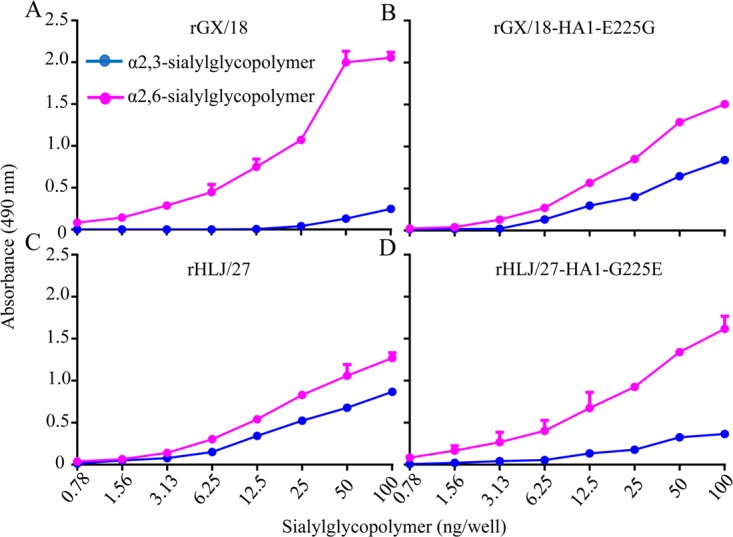
Characterization of the receptor-binding specificities of EAH1N1 SIVs. The binding of the viruses to two different biotinylated glycans was tested in a solid-phase binding assay. The data shown are the means of three replicates; the error bars indicate standard deviations.

Receptor-binding preference plays an important role in the replication and transmission of influenza viruses (18–20). Previous studies have shown that the amino acid at position 225 in HA1 is located in the receptor-binding site (10, 21, 22). We therefore evaluated the receptor-binding preferences of the rGX/18, rGX/18-HA1-E225G, rHLJ/27, and rHLJ/27-HA1-G225E viruses by using solid-phase binding assays, as described previously (5, 23, 24), with two different glycopolymers. All four viruses bound to the α2,6-glycopolymer (human-type receptor) with higher affinity than to the α2,3-glycopolymer (avian-type receptor) (Fig. 7), although the affinity of rHLJ/27 and rGX/18-HA1-E225G for the α2,3-glycopolymer was higher than that of rGX/18 and rHLJ/27-HA1-G225E. This result indicates that the E225G or G225E mutation in HA changes only the receptor-binding affinity and does not cause a switch in the receptor-binding preferences of the GX/18 and HLJ/27 viruses.

### The amino acid at position 225 of HA does not affect viral binding to A549 cells.

**FIG 8:**
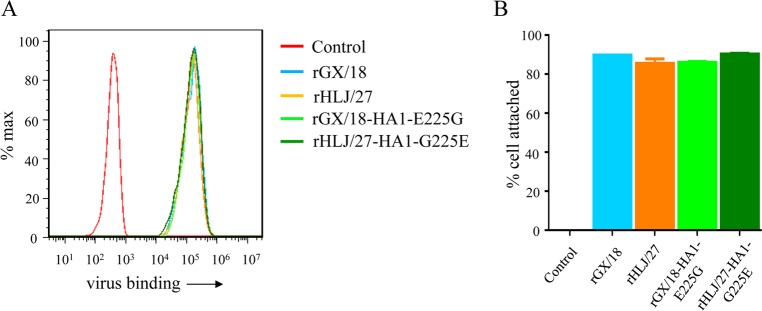
Assessment of the abilities of EAH1N1 SIVs to attach to the cell surface. A549 cells were seeded and inoculated at an MOI of 5 with four tested viruses. (A) Virus-bound and unbound cells were analyzed by flow cytometry. (B) Percentages of virus-bound cells as determined by using GraphPad software. The error bars indicate standard deviations.

Host cell attachment of influenza A virus is mediated by the HA protein. We therefore investigated whether the mutation at position 225 of HA influences viral binding to the cell surface. A549 cells were seeded and incubated with viruses at a multiplicity of infection (MOI) of 5 at 4°C for 1 h, and the virus-bound and unbound cells were analyzed by using flow cytometry. We found that the cells adsorbed similar amounts of the rGX/18, rHLJ/27, rGX/18-HA1-E225G, and rHLJ/27-HA1-G225E viruses (Fig. 8), indicating that the mutation at position 225 of HA does not affect viral attachment to the surfaces of A549 cells.

### The amino acid at position 225 of HA1 affects the assembly and budding efficiencies of virus-like particles.

**FIG 9:**
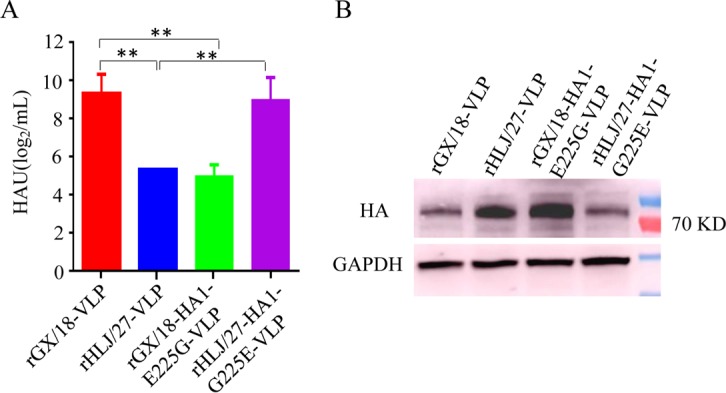
The amino acid at position 225 in HA affects the assembly and budding of virus-like particles. 293T cells were cotransfected with the pCAGGS-HA, pCAGGS-NA, and pCAGGS-M1 plasmids. (A) The supernatants were collected to purify the VLPs by sucrose density gradient centrifugation, and the hemagglutination titer was measured to detect the amount of VLPs. **, *P* < 0.01. The error bars indicate standard deviations. (B) The transfected cells were lysed to measure the amount of intracellular HA0 protein. GAPDH was used as the loading control.

HA initiates progeny virus particle assembly and budding through lipid raft domains in the apical plasma membranes of infected cells (25–27); assembling and budding efficiencies, in turn, affect influenza virus replication. Virus-like particles (VLPs) are widely used to assess the efficiency of influenza virus assembly and budding (28). We examined the effect of the amino acid at position 225 of HA on VLP formation by producing four different VLPs, designated rGX/18-VLP, rHLJ/27-VLP, rGX/18-HA1-E225G-VLP, and rHLJ/27-HA1-G225E-VLP, and comparing their productivities. We found that rGX/18-VLP and rHLJ/27-HA1-G225E-VLP produced significantly higher levels of hemagglutinin units in the cell supernatants than did rGX/18-HA1-E225G-VLP and rHLJ/27-VLP (Fig. 9A). We analyzed the HA levels of the four samples in the cell pellets by Western blotting and found that the levels of intracellular HA in GX/18 and rHLJ/27-HA1-G225E were considerably lower than those in HLJ/27 and rGX/18-HA1-E225G (Fig. 9B). These results indicate that the amino acid at position 225 of HA1 affects the assembly and budding of these two EAH1N1 viruses, which, in turn, affects their replication in cells and transmissibility in guinea pigs.

## DISCUSSION

In the present study, we explored the genetic basis for the difference in transmissibility in guinea pigs of two EAH1N1 swine influenza viruses, GX/18 and HLJ/27, and found that the single-amino-acid mutation E225G in HA1 abolishes the transmissibility of GX/18 and that the mutation G225E in HA makes HLJ/27 highly transmissible in guinea pigs. We further investigated the underlying mechanism and demonstrated that viruses bearing 225E in their HA1 replicate more rapidly than viruses bearing 225G in HA1 due to differences in their assembly and budding efficiencies.

HA is an important determinant for the transmission of the various subtypes of influenza virus, and several key amino acids in HA that are critical for this property have been identified (10, 11, 13, 18, 19, 29). Two amino acid mutations, D190E and D225G, abolished the ability of the 1918/H1N1 virus to transmit via respiratory droplet between ferrets (10). 226Q in HA is important for the transmission of the 2009/H1N1 virus in both guinea pigs and ferrets (11). The absence of glycosylation at residues 158 to 160 of the HA gene of H5N1 viruses is pivotal for their transmission in guinea pigs (13) and ferrets (19). All of these amino acid changes alter the transmissibility of influenza viruses by affecting their receptor-binding preferences. Imai et al. (18) reported that HA stability also plays an important role in virus transmission. They found that a reassortant virus containing seven genes from pH1N1/09 and the HA gene from an H5N1 virus could transmit between ferrets by acquiring HA-N224K/Q226L/T318I mutations. HA-N224K/Q226L switched the receptor-binding specificity from avian to human type but simultaneously reduced HA heat stability, which was restored by the HA-T318I mutation. Here, we found that the G225E mutation in HA increases the transmission of EAH1N1 viruses in guinea pigs by reducing virus affinity for the avian-type receptor and influencing the efficiency of virus assembly and budding. These findings indicate that the HA of influenza virus can affect its transmissibility through different mechanisms.

The rGX/18-HLJHA virus transmitted to one guinea pig, and the D24N mutation in HA1 occurred in the viruses that were recovered from the exposed animals in both experiments. Zhang et al. previously reported that amino acid 271A of PB2 plays a key role in virus acquisition of the mutation at position 226 of HA1 that confers human receptor recognition and transmission (11). It remains to be seen whether there are other viral factors in the GX/18 virus background that promote the acquisition of the D24N mutation in the HA1 of HLJ/27.

**FIG 10:**
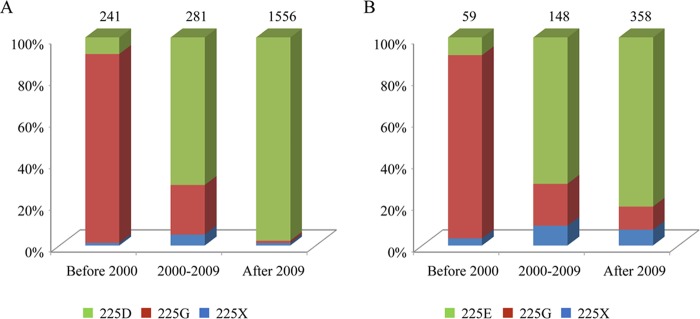
Amino acid at position 225 in the HA protein of naturally isolated swine influenza viruses. HA protein sequences of CSH1N1 SIV (without the delta lineage viruses) and EAH1N1 SIV (2,078 and 565, respectively) were downloaded from the National Center for Biotechnology Information. “X” denotes amino acids other than D and G, or E and G.

Two lineages of H1N1 SIVs, classical H1N1 SIVs and EAH1N1 SIVs, have been circulating in pigs since 1918 and 1979, respectively. The classical H1N1 SIVs emerged in humans as a reassortant and caused the 2009 H1N1 influenza pandemic, and the HA gene of 2009/H1N1 also belongs to the classical H1N1 SIV lineage. Our present study and a study by Tumpey et al. (10) indicate that 225E and 225D in HA1 play important roles in the transmissibility of EAH1N1 SIVs and classical H1N1 SIVs, respectively. We checked the HA sequence of the H1N1 SIVs and found that 225G was present in the HA1 genes of the majority of strains detected before the year 2000; however, the number of classical H1N1 SIVs that contain 225D in HA1 gradually increased after 2000 and became predominant after 2009 (Fig. 10A), and the EAH1N1 SIVs that contain 225E in HA1 showed a similar tendency (Fig. 10B). These findings suggest that the pandemic potential of the EAH1N1 SIVs is increasing.

## MATERIALS AND METHODS

### Ethics statement and facility.

The present study was carried out in strict accordance with the recommendations in the Guide for the Care and Use of Laboratory Animals of the Ministry of Science and Technology of the People's Republic of China. All studies were conducted in a biosecurity level 2 laboratory approved for such use by the Harbin Veterinary Research Institute (HVRI) of the Chinese Academy of Agricultural Sciences (CAAS). The protocol was approved by the Committee on the Ethics of Animal Experiments of the HVRI of the CAAS.

### Cells and viruses.

MDCK cells and human embryonic kidney cells (293T) were maintained in Dulbecco's modified Eagle's medium supplemented with 5% and 10% fetal bovine serum, respectively. The Eurasian avian-like H1N1 viruses A/swine/Guangxi/18/2011 (GX/18) and A/swine/Heilongjiang/27/2012 (HLJ/27) were isolated in slaughterhouses during routine surveillance from 2010 to 2013.

### Construction of plasmids for virus rescue.

The eight gene segments of GX/18 and HLJ/27 were inserted into the vRNA-mRNA bidirectional transcription vector pBD to rescue rGX/18, rHLJ/27, and all of the reassortant and mutant viruses as described previously (17). Mutations were introduced into the HA gene in the background of GX/18 and HLJ/27 by site-directed mutagenesis (Invitrogen) according to the manufacturer's protocol. All of the rescue viruses were completely sequenced to ensure the absence of unwanted mutations.

### Studies in guinea pigs.

Six-week-old Hartley strain female guinea pigs (Vital River Laboratories, Beijing, China) that were serologically negative for influenza viruses were used in these studies. During the experiments, the guinea pigs were housed in cages placed inside isolators. To examine virus transmissibility, groups of three guinea pigs anesthetized with ketamine (20 mg/kg of body weight) and xylazine (1 mg/kg) through intramuscular injection were inoculated intranasally with 10^6^ EID_50_ of the test viruses in a 300-μl volume (150 μl per nostril). Twenty-four hours later, three naive guinea pigs were placed in an adjacent cage (4 cm away) separated by a double-layer net divider. The ambient conditions were set at 20 to 22°C and 30 to 40% relative humidity. The airflow was horizontal with a speed of 0.1 m/s, and the direction was from the inoculated groups to the exposed groups. Nasal washes were collected at 2-day intervals, beginning on day 2 p.i. (day 1 postexposure), and titrated in embryonated chicken eggs. To avoid inadvertent physical transmission of virus by the investigators, all operational processes were from the exposed animals to inoculated animals, and all gloves, implements, and napkins on the worktop were changed between animals. Serum samples were collected from the guinea pigs on day 21 p.i., treated with Vibrio cholerae receptor-destroying enzyme (DenkaSeiken) for 18 h at 37°C, heat inactivated at 56°C for 30 min, and then tested for HI antibody titers by using 0.5% (vol/vol) chicken erythrocytes.

To investigate virus replication *in vivo*, groups of three guinea pigs were i.n. inoculated with 10^6^ EID_50_ of test virus in a 300-μl volume (150 μl per nostril). The animals were euthanized on day 3 p.i., and nasal washes and lungs were collected for virus titration in eggs.

### Receptor-binding analysis.

Receptor-binding specificity was analyzed by use of a solid-phase direct-binding assay with two different glycopolymers: α-2,3-sialylglycopolymer (Neu5Acα2-3Galβ1-4GlcNAcβ1-para-aminophenyl [pAP]-alpha-polyglutamic acid [α-PGA]) (avian-type receptor) and the α-2,6-sialylglycopolymer (Neu5Acα2-6Galβ1-4GlcNAcβ1-pAP-α-PGA) (human-type receptor), as described previously (18, 23). Guinea pig antiserum against GX/18, HLJ/27, and the reassortant and mutant viruses served as the primary antibodies, and a horseradish peroxidase (HRP)-conjugated goat anti-guinea pig antibody was used as the secondary antibody. Absorbance was determined at a wavelength of 490 nm. The cutoff value for the glycan binding assays was the background value of the well with 100 ng of glycopolymer in the absence of added virus.

### Viral replication in MDCK cells.

To evaluate virus growth kinetics, monolayers of MDCK cells were inoculated with viruses at an MOI of 0.01 and were maintained in Opti-MEM (Gibco, Grand Island, NY, USA) containing 0.2 g/ml l-(tosylamido-2-phenyl) ethyl chloromethyl ketone (TPCK)-treated trypsin (Sigma-Aldrich, St. Louis, MO, USA) at 37°C. Aliquots of culture supernatant collected from triplicate cultures at various time points (hours postinfection) were immediately frozen at −70°C until they were used.

### Viral attachment assay.

The ability of viruses to bind to the cell surface was evaluated by flow cytometry. Briefly, A549 cells were infected with viruses at an MOI of 5 at 4°C for 1 h. After three washes with PBS to remove unbound viruses, virus-bound cells were detected by adding an anti-HA rabbit polyclonal antibody (Sino Biological Inc.) for 1 h at room temperature. After hybridization with Alexa Fluor 488 donkey anti-rabbit secondary antibody (KPL), the cell suspensions were subjected to flow cytometry on a FACSAria flow cytometer (BD Biosciences, Franklin Lakes, NJ). The data were analyzed by using FlowJo software.

### VLP production and Western blot analysis.

pCAGGS-GX/18-HA, pCAGGS-GX/18-HA-E225G, pCAGGS-GX/18-NA, pCAGGS-GX/18-M1, pCAGGS-HLJ/27-HA, pCAGGS-HLJ/27-HA-G225E, pCAGGS-HLJ/27-NA, and pCAGGS-HLJ/27-M1 were constructed by inserting the corresponding cDNAs into the SacI/NheI sites of the pCAGGS vector. All the plasmids were completely sequenced to ensure their accuracy. Then, 293T cells grown in 10-cm dishes were cotransfected with 6 μg of HA plasmid, 6 μg of NA plasmid, and 12 μg of M1 plasmid by using the Lipofectamine 2000 transfection reagent (Invitrogen). After a 6-h incubation, the medium was replaced with Opti-MEM. At 60 h posttransfection, the supernatants were collected and VLPs were purified by means of sucrose density gradient centrifugation. Sediments were dissolved in 300 μl of PBS, and the VLP content was assessed by measuring hemagglutination titers. The cells were lysed in RIPA lysis buffer (Thermo; 25 mM Tris · HCl, pH 7.6, 150 mM NaCl, 1% NP-40, 1% sodium deoxycholate, 0.1% SDS) supplemented with 1× proteinase inhibitors, and the total protein concentration was evaluated by using a bicinchoninic acid (BCA) protein assay (Pierce). Equal amounts of total protein were fractionated by use of SDS-12% PAGE and transferred to nitrocellulose membranes (Pall). The proteins were then probed with either an anti-HA rabbit polyclonal antibody (Sino Biological Inc.) or an anti-GAPDH (glyceraldehyde-3-phosphate dehydrogenase) mouse monoclonal antibody (Proteintech) for 2 h at room temperature. After hybridization with goat anti-rabbit secondary antibody and goat anti-mouse secondary antibody (KPL), respectively, the membranes were visualized with enhanced chemiluminescence reagents (Pierce). All experiments were performed in triplicate to ensure reproducibility.
